# The role of the endocannabinoid system in managing neuropsychiatric symptoms in Alzheimer’s disease

**DOI:** 10.3389/fpsyt.2025.1709266

**Published:** 2026-01-13

**Authors:** Jagadeesh S. Rao, Maria Alejandra Tangarife, Maria Margarita Venegas, Barbara Forero, Laura Lucía González Tamayo, Juan Pablo Escobar-Gallego, Diego A. Rodríguez-Soacha, Alexandra Ferreiros, Ram Mukunda

**Affiliations:** IGC Pharma Inc., Potomac, MD, United States

**Keywords:** agitation, Alzheimer’s disease, anxiety, apathy, appetite, delusions, depression, hallucinations

## Abstract

The endocannabinoid system comprises cannabinoid receptors (CBRs) 1 & 2, endocannabinoids (eCBs) anandamide (AEA) and 2-arachidonoylglycerol (2-AG), and the enzymes that regulate their production and degradation. ECS plays a significant role in both health and disease. It influences neuronal and glial communications, neurotransmitter regulations, neuroinflammation, and behavioral alterations. Neuropsychiatric symptoms (NPS) are commonly seen in neurodegenerative conditions like Alzheimer’s disease (AD), apart from the core clinical diagnosis of dementia. NPS consists of various disturbing symptoms, including anxiety, agitation, apathy, hallucinations, delusions, sleeping problems, appetite problems, and depression. In AD, up to 97% exhibit one or more NPS. Emerging evidence from preclinical and clinical studies suggests that ECS is both a contributor to and a potential therapeutic target for managing NPS. This review explores ECS’s role in NPS and its therapeutic implications.

## 1 Endocannabinoid system

The endocannabinoid system (ECS) consists of two main cannabinoid receptors (CBRs) named CB1R and CB2R, and their natural endocannabinoids (eCBs), anandamide (AEA), and 2-arachidonoylglycerol (2-AG), and the enzymes responsible for their synthesis and breakdown (1). AEA is a partial agonist of both receptors, produced by the enzyme N-acyl phosphatidylcholine-specific phospholipase D (NAPE-PLD) and primarily degraded by fatty acid amide hydrolase (FAAH) (2). In contrast, 2-AG is a full agonist at both CB1R and CB2R, present at much higher levels than AEA (2–4). It is synthesized by diacylglycerol lipase (DAGL) and mainly broken down by monoacylglycerol lipase (MAGL), along with α/β-hydrolase domain-containing 6 (ABHD) 6, and ABHD12 (2, 5). CB1R is abundantly expressed throughout the brain and in several peripheral tissues, whereas CB2R is predominantly localized on immune cells in peripheral organs. Within the CNS, CB2Rs are mainly found on microglia, with only minimal expression in astrocytes and the brainstem (6, 7). Although CB1Rs are expressed on some immune cells, the ECS’s anti-inflammatory actions are primarily mediated via CB2R. Activation of CB2Rs in microglia plays a key role in controlling oxidative stress and dampening neuroinflammatory responses by promoting the release of anti-inflammatory mediators (8).

### 1.1 Dysregulation of the endocannabinoid system in AD

**Figure 1:**
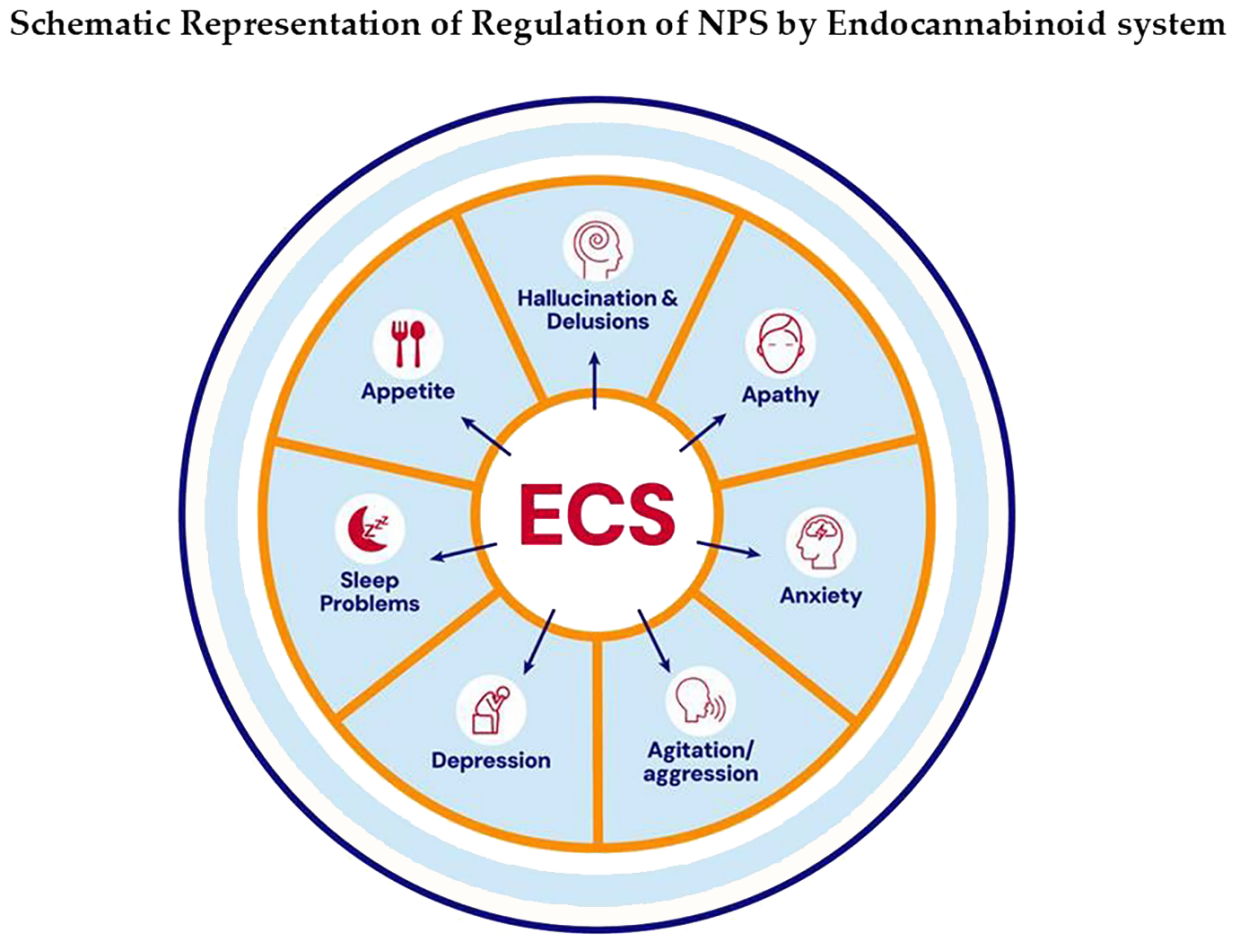
Altered ECS has been reported in both preclinical and clinical studies of AD. Modulators/agents targeting CBRs have been shown to have therapeutic effects on various NPS in AD. ECS participates in regulating many NPS in AD.

**Figure 2:**
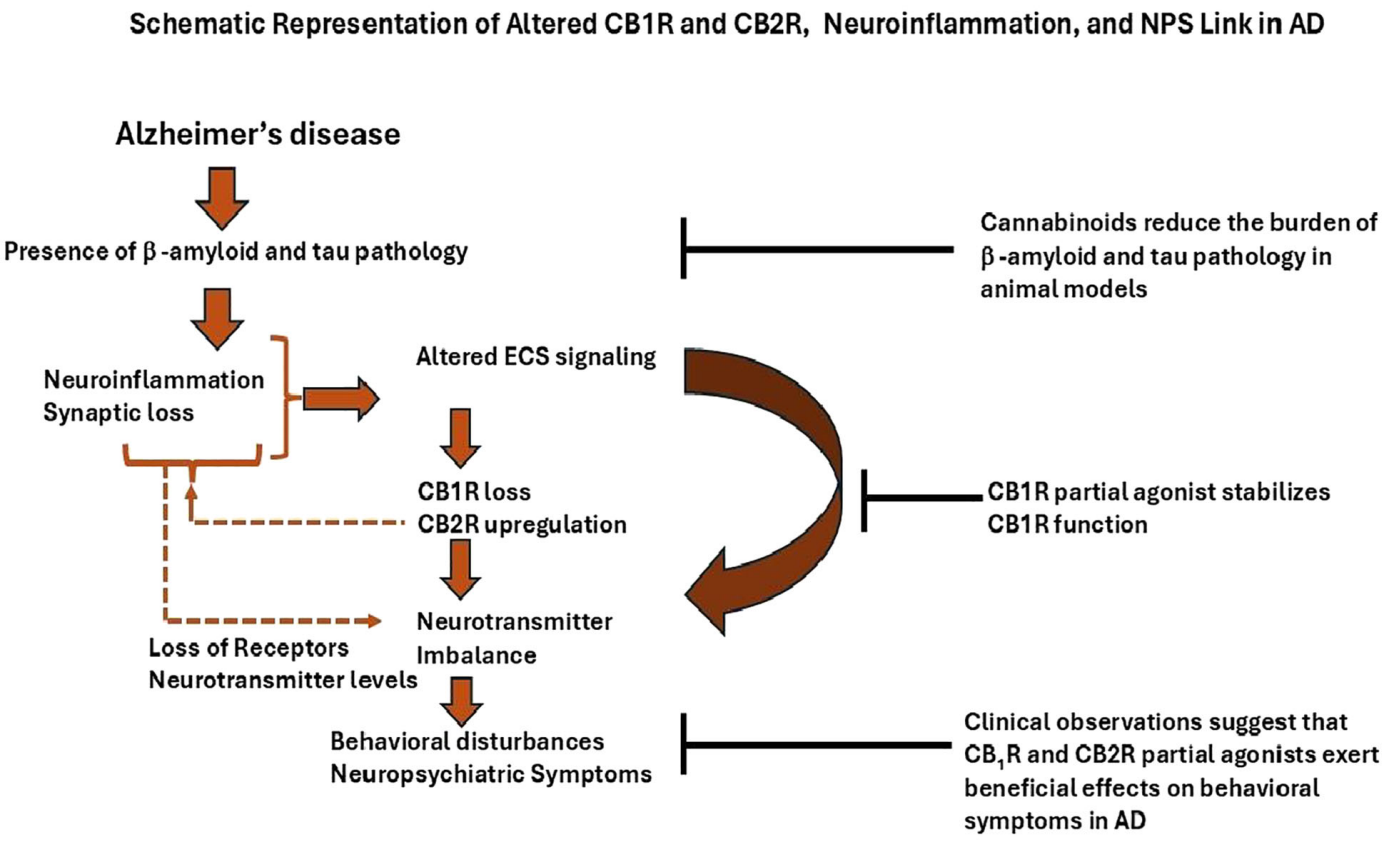
This schematic illustrates the proposed connection between neurotransmitter imbalance, neuroinflammation, and reduced activity of CB1R, which is related to aggression and other behavioral issues in AD. Decreased CB1R function is linked to disrupted GABAergic and serotonergic neurotransmission and increased neuroinflammatory signaling, and synaptic loss. Observational studies indicate that CB1R partial agonists, such as Δ^9^-tetrahydrocannabinol (Δ^9^-THC), could help alleviate these behavioral symptoms by restoring endocannabinoid imbalance and inhibiting neuroinflammation activation. Overall, these findings suggest that modulating ECS could be a potential therapeutic approach for addressing NPS in AD.

Alzheimer’s disease (AD) is the most common neurodegenerative disorder, characterized by a progressive decline in cognitive function and the frequent occurrence of neuropsychiatric symptoms (NPS) (9). It is currently the sixth leading cause of death in the United States and the fifth worldwide (10). Pathologically, AD is defined by two hallmark features: the extracellular accumulation of amyloid-β (Aβ) plaques and intracellular neurofibrillary tangles composed of hyperphosphorylated tau protein. Importantly, up to 97% of individuals with AD experience NPS at some point during the disease, placing a significant emotional and physical burden on caregivers (10). These symptoms include a range of behavioral and psychological disturbances such as apathy, depression, sleep disturbances, hallucinations, delusions, appetite problems, agitation, and disinhibition (11). Several preclinical and clinical studies on AD demonstrate that altered ECS contributes to pathology and behavioral alterations such as agitation, anxiety, apathy, sleeping disturbances, depression, disinhibition, hallucination, delusions, and its intervention by its receptor agonists has shown therapeutic effects (12–14) (**Figure 1**). Cognitive deficits observed in the AβPPswe/PS1ΔE9 AD mouse model are associated with decreased eCBs levels and increased cannabinoid receptor signaling activity in the striatum (15). Furthermore, the activation of cannabinoid receptors has been shown to reduce neuroinflammation in animal models of AD. For instance, a novel and selective CB2R agonist, *1-(3-benzyl-3-methyl-2,3-dihydro-1-benzofuran-6-yl) carbonyl piperidine* (MDA7), which lacks psychoactive effects, was found to attenuate neuroinflammation, synaptic dysfunction, and cognitive deficits induced by amyloid-β (Aβ 1–40) fibrils injected into the CA1 region of the hippocampus in rats (16). Similarly, prolonged oral administration of a synthetic CB1R agonist in Tg APP2576 mice reduced neuroinflammation, lowered Aβ levels, and improved cognitive performance (17). CB1R activation also facilitates the clearance of Aβ across the blood–brain barrier, suggesting a role in Aβ elimination (18) (**Figure 2**). Both CB1R and CB2R have been shown to reduce tau phosphorylation and promote neuroprotection (19) (**Figure 2**). In contrast, decreased CB1R expression in APP/PS1 transgenic mice is associated with worsened learning and memory deficits and reduced levels of PSD-95, a key postsynaptic density protein, highlighting the protective role of CB1Rs in AD pathology (20). Moreover, selective deletion of CB1R in the hippocampus leads to increased neuroinflammation, impaired cell proliferation, and deficits in social memory in adult mice (21) (**Figure 2**). Collectively, these findings support the therapeutic potential of CB1R modulation in the treatment of AD. Postmortem analysis of hippocampal brain tissue has revealed molecular alterations in the 2-AG signaling network, particularly near senile plaques (22). As AD progresses, overall CB1R density decreases, although higher levels are retained in specific regions, such as parts of the hippocampus and the deeper layers of the frontal cortex (15). In contrast, CB2R expression increases during the later stages of AD, coinciding with heightened activation of microglia and astrocytes and a marked rise in neuroinflammatory activity (23). This review examines the dysfunction of the ECS in the development of NPS and explores its potential as a therapeutic target for each symptom associated with AD.

## 2 Agitation

An earlier study reported that among NPS in AD, irritability/aggression had the highest frequency (76.2%), followed by apathy (72.3%) and depressive symptoms (68.0%) (24). More than 90% of individuals with AD exhibit one or more comorbid neuropsychiatric conditions (24). The prevalence of agitation in AD is estimated to range between 30% and 50%, while it is observed in about 30% of patients with dementia with Lewy bodies, and 40% in cases of frontotemporal and vascular dementia (25, 26). Agitation is typically characterized by verbal and physical aggression, including shouting, restlessness, and combative behavior, which significantly impair social relationships and activities of daily living (27). The underlying cause of agitation remains unclear; however, multiple pathological mechanisms have been implicated, including elevated neuroinflammation in specific brain regions, activation of the NOD-like receptor protein 3 (NLRP3) inflammasome, loss of CB1R function, and disruptions in neurotransmitter balance (28, 29). Recent positron emission tomography (PET) studies in AD patients have shown increased neuroinflammation in the medial temporal regions, which correlates with agitation symptoms (30). In preclinical models, activation of the NLRP3 inflammasome has been implicated in aggressive behaviors, suggesting a potential inflammatory mechanism underlying agitation (31). Additionally, loss of CB1R function has been linked to aggression in CB1R knockout mice, a phenotype that was reversed by acute administration of CB1R agonists (13). Notably, reduced CB1R activity in the frontal cortex and hippocampus has also been observed in postmortem brain tissue from AD patients (32). Furthermore, deficits in GABAergic and serotonergic neurotransmission have been associated with agitation in AD, suggesting that neurotransmitter imbalance contributes to the development of these behavioral symptoms (33). Recent studies have shown a stronger association between tau pathology and NPS, particularly agitation, than with β-amyloid (Aβ) deposition (34, 35). Functional imaging studies further support this by revealing dysregulation in the prefrontal cortex and subcortical regions, which are key areas implicated in the development of agitation in AD (36–38). Additionally, neuroinflammation in the medial temporal lobe and surrounding regions has been linked to the emergence of agitation symptoms in patients with AD (30). Importantly, neuroinflammation appears to be a response to Aβ and tau accumulation, rather than the primary cause of these proteinopathies (39). It also contributes to neurotransmitter imbalances (40) and synaptic loss (41). Both of which are central to the progression of behavioral symptoms in AD.

The ECS has been shown to regulate movement. CB1 receptors and endocannabinoids are highly expressed in the basal ganglia and have close connections with the dopaminergic system, which regulates motor functions. CB1R has been detected in subthalamonigral and subthalamopallidal terminals, glutamatergic cortico striatal afferences, and striatal projections to the *globus pallidus* and to the *substantia nigra (SN)* (1). Combined activation of CB1R and dopamine 1 receptors results in a decrease in the inhibitory activity of direct striatal projections and a decreased motor response. Conversely, activation of CB1R and D2 receptors potentiates the indirect pathway, resulting in motor inhibition (2). Lastly, ECS can indirectly modulate dopamine release in the SN (3).

Evidence of a biphasic effect of phytocannabinoids, synthetic cannabinoids, and endogenous cannabinoids on motor activity has been observed: high doses produce hypoactive effects, while low doses produce hyperactive effects (4). Resembling PD, Alzheimer’s patients can have loss of motor function, which can include reduced speed gait, loss of strength, reduced balance, and dexterity. In an AD animal model, treatment with cannabinoids (WIN 55,212-2) significantly improved performance in motor coordination, balance, and motor learning (4).

Therefore, regulating the ECS may help restore the balance between the direct and indirect pathways. This, in turn, could counteract the hyper- or hypodopaminergic state and the resulting increase/decrease in motor activity, thereby helping to improve aberrant motor behaviors, such as agitation or catalepsy, observed in patients with AD. Nonetheless, significant gaps remain in our understanding of motor disturbances in AD, particularly regarding their connection with the endocannabinoid system. Clarifying these mechanisms could open new avenues for therapeutic intervention. This is a promising field that urgently requires further exploration.

**Figure 3:**
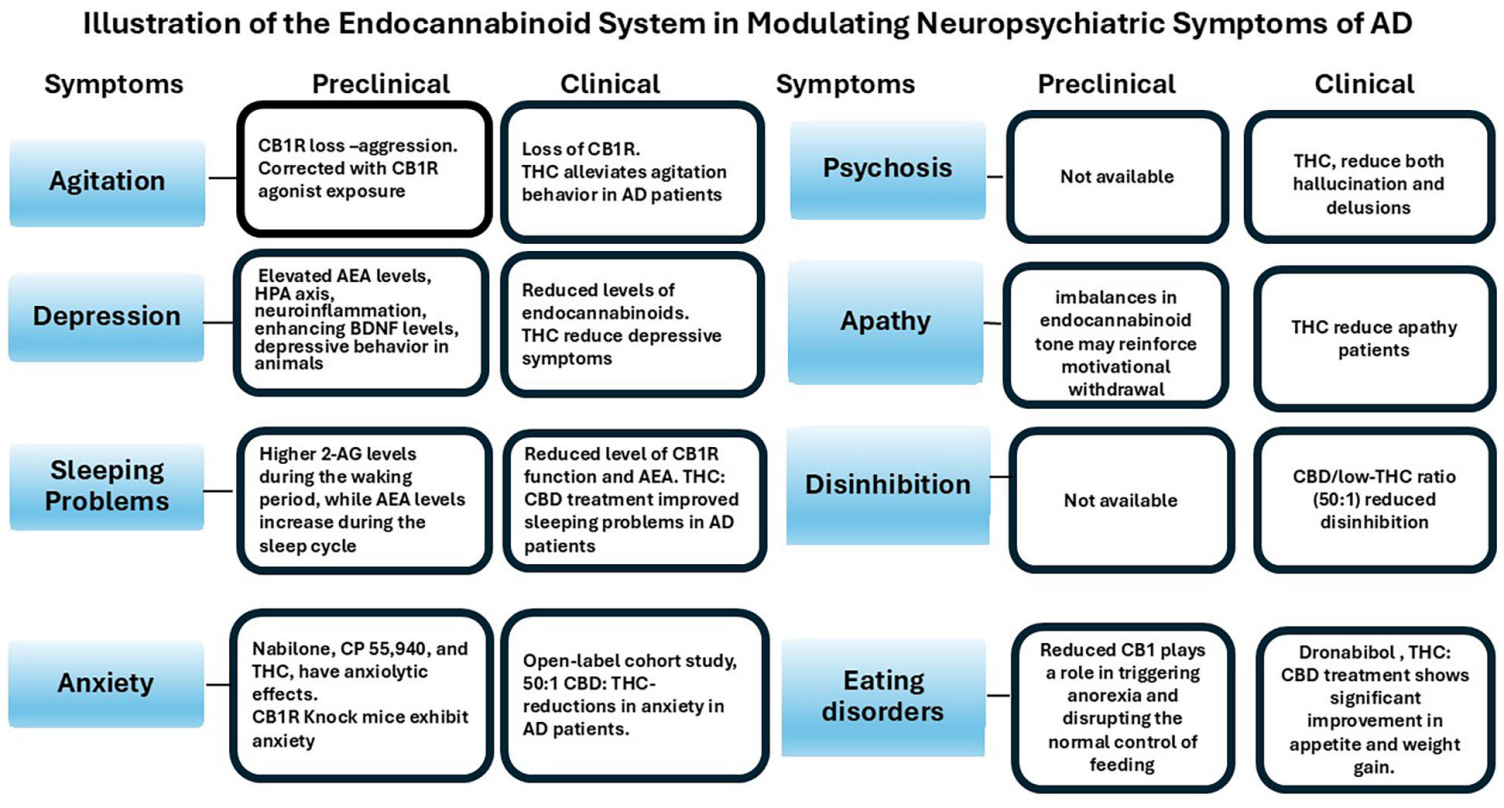
This schematic outlines the preclinical and clinical evidence connecting dysregulation of the endocannabinoid system (ECS) to significant NPS in AD. The ECS, which includes cannabinoid receptors (CB_1_R and CB_2_R), endogenous ligands (anandamide, 2-AG), and metabolic enzymes, plays a role in regulating neurotransmission, neuroinflammation, and emotional behavior. Preclinical studies suggest that decreased CB_1_R signaling is linked to increased aggression and anxiety, while cannabinoid agonists are found to mitigate these behaviors. In clinical settings, the loss of CB_1_R in the frontal cortex and hippocampus in AD, along with the use of agonists for CB_1_R and CB_2_R, has been shown to alleviate related symptoms. Changes in endocannabinoid tone are associated with disturbances in mood, sleep, and anxiety, with formulations that are CBD-dominant or low in THC demonstrating symptomatic relief. Partial agonists of CB_1_R and CB_2_R, such as Δ^9^-THC, have been found to decrease symptoms of psychosis and apathy, while synthetic cannabinoid treatments like dronabinol have a positive impact on appetite and weight management. Overall, the available evidence supports the notion that modulating the ECS could serve as a potential therapeutic strategy for addressing various NPS in AD, underscoring the necessity for larger, controlled clinical studies.

The ECS modulates behavior through multiple receptor pathways, particularly via CB1R. In AD patients, reduced CB1R expression in the hippocampus and frontal cortex has been reported, which may contribute to behavioral disturbances, including agitation (32, 42). Preclinical studies demonstrate that low doses of delta-9-tetrahydrocannabinol (THC) (0.2–2 mg/kg body weight) produce a dose-dependent anti-aggressive effect in mice, rats, and squirrel monkeys, particularly in intruder-based behavioral tests (43) (**Figure 3**). The ECS also plays a crucial role in modulating the release and function of various neurotransmitters, including GABA, serotonin (5-HT), and norepinephrine. Deficits in GABAergic and serotonergic signaling have been implicated in the pathophysiology of agitation in AD (33). Notably, CB1R activation has been shown to enhance [³H]-GABA release in rats (44), suggesting that CB1R agonists may help restore inhibitory tone and improve behavioral symptoms in AD. In addition, CB1R activation induces functional desensitization of postsynaptic α_2_-adrenergic receptors (α_2_-ARs) on layer V/VI pyramidal neurons in the medial prefrontal cortex (mPFC) (45), and has also been shown to inhibit serotonin reuptake *in vitro*, further supporting its role in neurotransmitter regulation and behavioral modulation (46).

Previous open-label clinical trials have indicated that the synthetic form of THC, dronabinol, may improve appetite, body weight, and nocturnal motor activity, and reduce agitation in patients with AD (47, 48). In severely demented patients, dronabinol treatment led to significant reductions in all domains of the Pittsburgh Agitation Scale, along with improvements in Clinical Global Impression (CGI) scores, sleep duration, and meal consumption. Although some adverse events were reported, none resulted in treatment discontinuation (49). A randomized, placebo-controlled trial of nabilone, a synthetic analog of THC and partial CB1R agonist, also demonstrated efficacy in reducing agitation, with additional benefits on overall NPS, caregiver burden, and nutritional status (50). Similarly, an open-label add-on trial using low doses of medical cannabis oil containing THC significantly reduced scores on the Neuropsychiatric Inventory (NPI) and clinical global Impression (CGI) -Severity scale in AD patients (14) (**Figure 3**). These findings support the therapeutic potential of CB1R activation in managing NPS, particularly agitation, in AD. Ongoing placebo-controlled randomized trials are further investigating the efficacy of THC in this context, including NCT02792257 (now completed, results pending) and NCT05543681. The outcomes of these studies are expected to provide important insights into the clinical utility of CB1R partial agonists for the treatment of behavioral symptoms in AD.

Major limitations of existing trials include the absence of randomized, placebo-controlled designs and inadequate sample sizes that were not statistically powered for predefined endpoints. Additionally, variability in behavioral rating scales used to assess NPS introduces inconsistency across studies. Patient recruitment across heterogeneous stages of AD further complicates the interpretation of treatment effects. In several studies, statistical analyses were insufficient to robustly evaluate clinical outcomes, and dose titration protocols did not consistently follow standards established for randomized controlled trials. Collectively, these methodological limitations restrict the generalizability and strength of conclusions regarding the efficacy of cannabinoid-based interventions for behavioral symptoms in AD. More systematic placebo-controlled randomized trials are further investigating the efficacy of THC in this context, including NCT02792257 and NCT05543681. Preliminary data from the NCT02792257 clinical trial indicate that dronabinol treatment was associated with a reduction in agitation symptoms (2024 Clinical Trials on Alzheimer’s Disease (CTAD) conference). The outcomes of these studies are expected to provide important insights into the clinical utility of CB1R partial agonists for the treatment of behavioral symptoms in AD. The findings of preclinical and clinical studies on the ECS’s role in aggression/agitation in AD were presented in **Tables 1** and **2**.

**Table 1:** ECS role in NPS regulation in animal models.

Behavioral symptoms	Animal model	Cellular alterations	Reference
Aggression	Resident intruder model	Increase in Inflammasome -3	(31)
Aggression	CB1R knock out	Loss of CB1R and aggressive behavior	(13)
Aggression	intruder-based behavioral tests	THC, a partial agonist to CBR1, produced dose-dependent anti-aggressive effects in mice, rats, and squirrel monkeys.	(43)
Depression/Neuroinflammation-associated behavioral changes	Stress-induced depressive model	Stress reduces CB1 receptor function and AEA levels, contributing to depressive behaviors	(52, 68)
Depression	Animal Model of Depression	Inhibiting FAAH and MAGL enhances endocannabinoid signaling, promoting hippocampal neurogenesis, synaptic protein expression, and reducing depression like symptoms	(71)
Anxiety	The elevated plus maze and light and dark tests	Low doses of CB1R agonists, such as nabilone, CP 55,940, and THC found to have anxiolytic effects in the elevated plus maze and light and dark tests	(113, 114)
Anxiety	Animal model	High doses of CB1R agonists produce anxiogenic behavior	(114)
Anxiety	CB1-KO mice	CB1-knockout mice displayed increased anxiety in the elevated plus-maze test, while administration with the CB1 antagonist SR141716A unexpectedly reduced anxiety in both wild-type and knockout mice.	(115)
Sleep Disorders	Randomized crossover trial comparing 4 nights of normal (8.5h) vs. Restricted sleep (4.5h) in healthy adults. Circulating concentrations of 2-AG and its structural analog 2-oleoylglycerol (2-OG) were measured every 15 to 30 minutes for 24h.	Under both normal sleep and restricted sleep conditions, 2−AG levels were reduced in the middle of the overnight fast and then gradually rose to a peak in the early afternoon. Sleep restriction amplified this rhythm, leading to a later and more sustained peak of 2−AG concentrations. Similarly, 2−OG levels follow this pattern, but with a smaller amplitude.	(90, 100)
Sleep Disorders	Wistar rats	Intrahippocampal administration of AEA in Wistar rats increased REM sleep during the dark cycle, but it was blocked by the CB1R antagonist AM251.	(98, 99)
Apathy	Transgenic mouse model (PK−/−/Tau VLW) treated with Sativex^®^ (THC + CBD) daily for 1 month, starting at symptom onset	Reduced dopamine metabolism and oxidative stress; decreased gliosis, iNOS expression, tau phosphorylation, and Aβ plaque/oligomer load; increased GSH/GSSG ratio and autophagy	(137–139)
Apathy/Excitotoxicity-related symptoms	*In vitro* NMDA-induced excitotoxicity in murine cortical neurons; *in vivo* NMDA-lesion model in CB1R WT and knockout mice	CB1R activation reduced NMDA-induced neuronal death via inhibition of NO production and PKA signaling; CB1R deletion increased NOS activity and lesion size	(142)
Eating Disorders (Anorexia)	Male Wistar rats	Activation of CB1 receptors in the paraventricular nucleus inhibited 5-HT release (decreasing serotonergic activity via 5-HT1A/1B) and disinhibited GABA release, resulting on increased food intake. Effect blocked by CB1 antagonist (AM251) and by 5-HT1A/1B agonists.	(164)

**Table 2:** ECS role in NPS regulation in clinical studies in humans.

Behavioral symptoms	Study model-design	Cellular alterations - results	Reference
Agitation	Alzheimer’s patient	Dronabinol treatment: Improvement with Appetite, body weight, nocturnal motor activity, and reduction in agitation in patients with AD	(46, 47)
Agitation and Sleep	Alzheimer’s Patient	A significant reduction in all domains of the Pittsburgh Agitation Scale, along with improvements in Clinical Global Impression (CGI) scores, sleep duration, and meal consumption	(48)
Agitation	Alzheimer’s Patient	Nabilone, a partial agonist to CB1R, demonstrated efficacy in reducing agitation	(50)
Depression/Dependence-related neuroadaptations	Human PET imaging study in chronic daily cannabis smokers, with baseline and post-abstinence comparison over ~4 weeks	Region-specific downregulation (~20%) of CB1 receptors in cortical areas (neocortex and limbic cortex), reversible after abstinence	(66)
Depression/Anhedonia	Double-blind, placebo-controlled, parallel-group study in healthy human volunteers treated with CB1 antagonist (rimonabant) for 7 days: fMRI during reward/aversion tasks	Decreased neural activation to rewarding chocolate stimuli in ventral striatum and orbitofrontal cortex; altered responses to aversive stimuli in caudate and lateral orbitofrontal cortex	(69)
Anxiety	Alzheimer’s Patient	50:1 CBD: THC: reductions in anxiety, agitation, and aggression	(116)
Sleep Disturbances	Alzheimer’s Disease Patient	Decreased AEA, disturbed CB1/CB2 signaling at the BBB, and reduced astrocyte function may contribute to some of the characteristic sleep disturbances observed in AD	(106)
Psychosis	AD	A partial agonist of CB1R and CB2R, derived from cannabis, such as delta-9 THC, has been found to reduce both hallucination and delusions	(14, 116)
Eating Disorders (Anorexia)	Fifteen patients with AD; double-blind placebo-controlled crossover trial, 6-week periods of dronabinol (2.5 mg BID) vs placebo. 12 patients were included in the analysis.	Dronabinol led to significantly greater weight gain, improved triceps skinfold thickness, and reduced Cohen-Mansfield Agitation Inventory (CMAI) scores. Negative effects also decreased more with dronabinol.	(47)
Eating Disorders (Appetite loss)	Thirty AD patients, mild–severe AD, observational retrospective trial; oral cannabis oil extract (22% THC, 0.5% CBD in olive oil), 0.5–1 ml/day titrated, given sublingually twice daily for 12 weeks.	Oral cannabis extract led to a significant reduction in NPI-Q scores (including eating disturbances); 70% of patients had an increase in appetite and weight gain.	(156, 159)

## 3 Depression

Depression and AD are two chronic conditions that share several pathophysiological mechanisms, including neuroinflammatory processes, disruptions in neurotrophic signaling cascades, structural abnormalities in dendritic spines, and disturbances in neurotransmitter homeostasis (51). In recent years, the ECS has been explored as a promising therapeutic area and a potential link between depression and depression leading to AD (8).

Major depressive disorder (MDD) is a psychiatric disorder characterized by a loss of interest and pleasure (anhedonia), as well as decreased performance in daily activities. It usually includes symptoms such as hopelessness and reduced psychosocial functioning (52). Contemporary definitions of depression not only consider its affective and motivational disturbances, but also its neurovegetative, cognitive, and psychomotor changes, reflecting the dynamic and heterogeneous nature of depression (53, 54).

The interplay between major depression and AD is well established. Individuals with a history of depression are more likely to develop AD later in life, positioning depression as a potential risk factor for the disease (55, 56). Evidence from the longitudinal, population-based Framingham Heart Study, which followed both depressed and non-depressed participants over 17 years, revealed that individuals with depression (mean age 81 ± 6 years) had a 1.5-fold increased risk of developing AD compared to their non-depressed counterparts (mean age 79 ± 5 years) (57). Further, depressive episodes and their associated symptoms contribute to greater rates of institutionalization, higher morbidity and mortality, and more pronounced behavioral disturbances in individuals with AD, ultimately accelerating functional decline (55, 58). Notably, over 50% of individuals with AD experience depression or depressive symptoms, and 20–30% go on to develop MDD (58).

A cohort study of 158 patients with late-onset AD found that polymorphisms in serotonin receptors 5-HT2A and 5-HT2C were associated with the development of depression (59). Patients with both depression and AD experience earlier and more severe cognitive decline, greater neuropathological changes, and increased loss of serotonin receptors and transporters compared to those with AD alone (60, 61). Analysis of brain samples from the U.S. National Alzheimer**’**s Coordinating Center revealed more advanced neurofibrillary tangle (NFT) stages in patients with both depression and AD than in those with only AD (62). Additionally, a two-year study of 282 Norwegian outpatients with AD showed that moderate and worsening depressive symptoms were linked to faster dementia progression (63). A recent cohort study shows that people with Alzheimer**’**s or other dementias have over twice the risk of developing depression, with the risk especially high in women and those over 85, indicating a lasting neuropsychiatric burden as the disease progresses (64).

Recent research indicates that dysregulation of the ECS contributes to the pathophysiology of depression, particularly through altered CB1R and CB2R signaling, impaired neurogenesis, and disrupted stress response mechanisms. CB1Rs are abundant in mood-regulating brain regions (e.g., prefrontal cortex, hippocampus, amygdala, and nucleus accumbens) (52, 65). Preclinical studies indicate that increasing anandamide levels through FAAH inhibition can reduce depressive behaviors, while modulation of the CBR1 and CBR2 receptors affects neuroplasticity. Clinically, CB1R antagonists have been linked to mood disturbances, and endocannabinoid deficits are observed in depressed patients (**Figure 3**). Additionally, genetic variations in CB1/CB2 genes are associated with depression risk and differential antidepressant responses (8, 66). The ECS also regulates neurotransmitter release, synaptic plasticity, and neurogenesis, which is particularly relevant in AD, where ECS-related neurotransmitter imbalances (serotonin, glutamate, GABA, acetylcholine) contribute to depressive symptoms via neuroinflammation, excitotoxicity, and synaptic dysfunction (51).

CB1R activation suppresses glutamate release, helping maintain synaptic homeostasis and reducing excitotoxicity under stress. However, chronic activation may reduce CB1Rs, potentially leading to depressive-like behaviors (67, 68). Despite this, ECS-mediated inhibition of neurotransmitter release can enhance dopamine function and motivation, as shown by studies linking CB1 receptor blockade to anhedonia (68, 69).

CB2Rs, though primarily found in peripheral immune cells, are also present in the CNS and play a role in neuroimmune regulation, especially under inflammatory conditions (65, 70). ECS signaling via CB2 receptors helps modulate immune responses by reducing pro-inflammatory cytokines and limiting T-cell proliferation. AEA and 2-AG influence inflammation through eicosanoid pathways, with AEA enhancing cognitive flexibility, while excess 2-AG may impair it (8, 68). Microglial and lymphocyte activation alter ECS function by affecting receptor expression and endocannabinoid metabolism. In aging and depression, hippocampal CB1R expression and 2-AG levels decline, and in AD, CB1R expression decreases while CB2 increases. These findings highlight the therapeutic potential of ECS modulation in treating depression and AD (51)(**Figure 3**).

Stress is a shared risk factor in both depression and AD, affecting the ECS and amyloid pathways. Chronic stress reduces CB1R function and AEA levels, contributing to depressive behaviors (52, 68). It also disrupts 2-AG metabolism via enzymes like MAGL, impairing synaptic plasticity and stress response (52, 71). In AD, stress and aging promote the formation of toxic Aβ42 oligomers, leading to oxidative damage and tau pathology (72). Depression is linked to increased cortical Aβ, while AD shows both excess production and poor clearance. Cannabinoids may counter these effects by modulating Aβ dynamics and enhancing microglial clearance, underscoring the ECS**’**s role in both disorders (51).

Neuroinflammation, driven by glial activation, cytokine release, and blood-brain barrier (BBB) disruption, is a key mechanism in both depression and AD (73). ECS regulates brain immune responses, with CB2 receptors playing a central anti-inflammatory role (74). In AD, CB2 and FAAH are overexpressed near amyloid plaques, and CB2R activation shifts microglia toward an anti-inflammatory state, reducing IL-1β, IL-6, and TNF-α while increasing IL-10 and BDNF, thereby protecting the BBB (75, 76). CB1Rs influence BDNF expression; acute activation increases BDNF, whereas chronic activation desensitizes CB1Rs and lowers BDNF. Since BDNF is reduced in depression and dysregulated in AD, altered ECS signaling likely contributes to neuroinflammation, impaired BDNF regulation, and depressive symptoms in AD (51).

Beyond stress and inflammation, the ECS also plays a vital role in neuroplasticity and mood regulation. It modulates the HPA axis, particularly through CB1R signaling in the amygdala, which is disrupted by stress via increased FAAH activity and reduced eCBs (74). Inhibiting FAAH and MAGL enhances endocannabinoid signaling, promoting hippocampal neurogenesis, synaptic protein expression (e.g., BDNF), and reducing depressive-like behaviors and inflammation (71). These effects also support dendritic spine stability, which is compromised in depression and AD. ECS dysregulation through CB1R downregulation or CB2R loss-of-function contributes to excitotoxicity and mood symptoms. Thus, targeting ECS metabolism offers therapeutic potential for depression and mood-related symptoms in AD (8, 51). **Tables 1** and **2** summarize key findings from preclinical and clinical studies examining the mechanisms and therapeutic approaches targeting depression in AD.

## 4 Sleeping disorders

Sleep disorders are defined as disruptions in the normal sleep process, including difficulty falling or staying asleep, reduced total sleep time, frequent nighttime awakenings, early morning awakenings, and excessive daytime sleepiness (77). Based on a meta-analysis performed by Koren et al. in 2023, it is reported that nearly 26% of AD patients experience sleeping problems (77). Historically, sleep problems have been viewed as symptoms secondary to neuropsychiatric conditions; however, these disturbances are now recognized as both early indicators and potential contributors to neurodegenerative diseases, including AD. Sleep quality naturally declines with age, and disruption of sleep architecture, particularly in deep sleep and rapid eye movement (REM) sleep, is a frequent antecedent of cognitive decline and the progression of AD (78, 79). Emerging evidence suggests that poor sleep may accelerate AD pathology by promoting amyloid-β accumulation, tau hyperphosphorylation, and neuroinflammation, and thereby contributing to faster neurodegeneration (80). These biological changes are linked not only to cognitive decline but also to worsening NPS, including agitation, aggression, and irritability (81, 82). One of the most consistent alterations in AD is the reduction in slow-wave sleep (SWS or N3, non-REM sleep), which plays a critical role in memory consolidation and glymphatic clearance (83, 84). Additional abnormalities include increased light (N1) sleep, REM latency, greater wakefulness after sleep onset (WASO), and reductions in sleep efficiency, total sleep time, and REM sleep (85). Many of these architectural features have been correlated with increased nighttime blood pressure, sleep hypoxia, and disruptions in the glymphatic system, which have been related to increased amyloid and other toxins in the interstitial space (84–87). These disruptions in sleep architecture are also associated with dysfunction in brain regions central to circadian and sleep regulation, such as the suprachiasmatic nucleus of the hypothalamus, the hippocampus, and the cortical gray matter of the frontal and temporal lobes (88).

The endogenous ligands of the ECS, mainly AEA and 2-AG, play a role in the modulation of sleep-wake cycles by fluctuating their levels across the day (89, 90). In brain regions associated with learning, memory, and motor control, such as the nucleus accumbens, prefrontal cortex, striatum, and hippocampus. 2-AG and AEA exhibit opposing diurnal patterns: 2-AG levels are higher during the waking period, while AEA levels increase during the sleep cycle (91–93)(**Figure 3**). Thus, indicating that 2-AG may be more related to promoting wakefulness, while AEA is for sleep (92, 94–96). Other studies suggest that AEA, via activation of CB1R, is associated with increased SWS. This has been supported by studies in animal models, where the exogenous administration of CB1R agonists reduced wakefulness and increased both NREM and REM sleep, while CB1R antagonists generated the opposite effect (97, 98). The activation of CB1R, through AEA, enhances the release of GABA, contributing to the induction of sleep and the maintenance of NREM sleep stages (89). On the other hand, 2-AG is increased during REM sleep, linking its role in modulating dreaming and cognition (99). However, it has also been regulated in contexts of sleep deprivation (100). Perez-Morales and colleagues (99) identified that direct administration of 2-AG into the lateral hypothalamus of mice increased the duration of REM sleep by increasing the concentration of melanin-concentrating hormone (MCH). This supports the notion that the ECS balance between excitatory and inhibitory inputs within these neural circuits enables the initiation and maintenance of wakefulness during the day and various stages of sleep, such as REM sleep at night.

In the context of AD, sleep disturbances are marked by fragmented sleep, reduced slow-wave (NREM) and REM sleep, elevated expression of proinflammatory cytokines, astrocyte reactivity, and impaired clearance of neurotoxic proteins via the glymphatic system (85, 101–103).

The ECS has been implicated in sleep dysregulation in AD. Available literature indicates that CB1R is decreased in AD and downregulated in regions including the hippocampus, neocortex, basal ganglia, and brainstem (42). Enzymes such as FAAH are upregulated, potentially lowering AEA’s availability to induce sleep and SWS (104). Alongside reduced SWS, sleep disturbances, and neuroinflammatory states in AD alter ECS activity in astrocytes, impairing sleep architecture and clearance of amyloid β (101, 105, 106). Because the CB1 receptors are located on the astrocyte end feet at the BBB (107). They modulate some of the mechanisms by which astrocytes modulate extracellular fluid and glymphatic clearance, influencing ion balance and neurotransmission at tripartite synapses, which are essential for normal sleep–wake transitions (105). Considering these findings, ECS dysregulation, particularly decreased AEA, altered CB1R/CB2R signaling at the BBB, and impaired astrocyte function, may contribute to some of the characteristic sleep disturbances observed in AD (105).

There is a small but growing body of evidence supporting cannabinoid therapy for sleep disturbances in AD. In a two-week open-label study of severe dementia, dronabinol 2.5 mg nightly reduced nocturnal motor activity on actigraphy and improved NPI night-time behaviors, including agitation (48). In a 16-week randomized trial, a cannabidiol (CBD)-rich oil (Avidekel^®^) significantly reduced behavioral disturbances versus placebo, with mostly non-serious adverse events; sleep-related NPI-NH items also improved (108). An observational 12-week cohort receiving twice-daily THC: CBD oil showed within-group reductions in NPI-Q sleep-disturbance scores alongside improvements in other NPS (12). Likewise, a prospective open-label cohort using a magistral cannabis formulation reported NPI-Q improvements across domains, including night-time behaviors (12). Taken together, these studies suggest potential sleep benefits in AD, but the evidence is limited by small samples and heterogeneity in formulations. **Tables 1** and **2** provide an overview of previous preclinical and clinical evidence from ECS on sleeping disorders in AD.

## 5 Anxiety

Anxiety is a frequent behavioral disturbance in AD, often appearing early and persisting throughout the illness (10). The prevalence of anxiety in AD is about 35% to 40% (109). Neuroimaging and pathology studies link anxious symptoms to core disease processes. Patients with anxiety more often show positive amyloid PET scans, atrophy, and hypometabolism in the mesial temporal lobe, especially the entorhinal cortex, and a high burden of neurofibrillary tangles (10). Biochemical evidence points in the same direction: higher cerebrospinal fluid t-tau/Aβ42 ratios, a marker of AD pathology, correlate with greater anxiety in people with mild cognitive impairment or Alzheimer**’**s dementia (11). From a clinical perspective, the presence of anxiety in AD significantly impacts patients**’** quality of life, increases caregiver distress, and is associated with higher rates of hospitalization, institutionalization, and mortality (34).

Alterations in the ECS may play a role in the high prevalence of anxiety observed in AD. CB1Rs are widely distributed throughout the brain, with high expression on GABAergic interneurons and additional presence on glutamatergic, cholinergic, glycinergic, and serotonergic neurons (35). Modulating this system can have either anxiolytic or anxiogenic effects, depending on the brain region involved, receptor subtype, and baseline neural activity (36). In AD, CB1R signaling contributes to the regulation of synaptic transmission, an area affected by the disease, and may offer neuroprotective effects (37, 38). Given these mechanisms, along with the appetite-stimulating and anxiolytic properties of certain cannabinoids, there is a strong rationale for investigating cannabinoid-based therapies in individuals with AD (38).

Several preclinical studies indicate that low doses of CB1R agonists, including both synthetic and natural compounds such as nabilone (mice; 10 μg/kg i.p.) (110), CP 55,940 (Rats; 10, 20, and 40 μg/kg) (111), and THC (mice; 0.3–5 mg/kg i.p.) (112), have anxiolytic effects in the elevated plus maze and light**/**dark tests (113, 114) (**Figure 3**). Whereas high doses of CB1R agonists produce anxiogenic effects in animal models (114). An animal model revealed that CB1-knockout mice showed increased anxiety in the elevated plus-maze test, while treatment with the CB1 antagonist SR141716A unexpectedly reduced anxiety in both wild-type and knockout mice. These results suggest that a novel CB1R may be involved in anxiety regulation (115). Overall, these studies suggest that the involvement of CB1R is in the regulation of anxiety-like behavior in animals.

Observational research suggests that cannabis formulations with a high CBD to low THC ratio may offer therapeutic benefits for managing anxiety and other NPS in patients with AD. In an open-label cohort study, a 50:1 CBD: THC formulation was associated with reductions in anxiety, agitation, and aggression in AD patients, as well as a decrease in caregiver burden (**Figure 3**). These effects were independent of patient age, disease duration, or prior use of acetylcholinesterase inhibitors. The observed anxiolytic benefits may be linked to activation of serotonin (5-HT) 1A receptor, alongside secondary effects such as improved sleep and pain relief (116). **Tables 1** and **2** summarize the key preclinical and clinical findings related to anxiety in AD.

## 6 Psychosis

Psychosis in neurodegenerative disorders encompasses hallucinations and delusions. It belongs to the NPS but is also a source of other things, like anxiety, depression, and agitation (117–120). Furthermore, the presence of psychosis is strongly associated with faster rates of cognitive decline and a higher risk of institutionalization (116, 117, 121). Hallucinations can be experienced through all senses, without a sensory stimulus. Usually, the most frequent type in AD patients is visual hallucinations, and they can range from only images to complex features or scenes; 13% to 18% of AD patients experience them (117, 118, 121). All hallucinations may share a common neural network that involves the right superior temporal sulcus, and based on the content, the connection of this network and the sensory modality; also, the presence of hallucinations might be associated with atrophy of the anterior right insula, superior frontal gyrus, lingual gyrus, and occipital lobes (117, 120, 122).

Delusions are strong but false beliefs held by patients that persist despite irrefutable evidence; they cover a spectrum from odd, curious, or unconventional to repetitive and mundane (117, 118, 121, 123). The prevalence of delusions in AD patients ranges from 16% to 70%, 35% to 36% on average (117, 118, 121), and over 44% patients with AD can present delusions associated with hallucinations, considering that hallucinations are often experienced with delusions rather than alone (118, 119, 121). The neural basis of delusions in AD has been linked to atrophy in medial temporal lobe structures, especially in cases of misidentification delusions, along with widespread disruption of the default mode network. There are over 14 subtypes of delusions, like grandiosity, guilt, jealousy, religious, thinking control-related, and more, but those most common in AD are related to paranoia, the sensation of being persecuted, and misidentification, this last one being a prevailing category in AD and other dementia syndromes (120, 121, 124). Studies of psychosis in AD described greater hypometabolism in the frontal and prefrontal cortex, and delusions are a significant predictor of amyloid plaque pathology, which correlates with findings that subjects with mild cognitive impairment who are amyloid-positive have a major occurrence of agitation, delusions, and hallucinations (120, 125).

ECS has been implicated in psychosis in schizophrenia, with a reduction in CB1R availability (126). Similarly, the reduced CB1R has been reported in postmortem brains from AD (32). These changes may contribute to the psychotic behavior in AD. In support of this notion, a partial agonist of CB1R and CB2R, derived from cannabis, such as delta-9 THC, has been found to reduce both hallucination and delusions (14, 116) (**Figure 3**). Activation of CB1Rs with agonists may help counteract receptor loss observed in AD, offering potential therapeutic benefits. The elevated expression of CB2R in the glial cells in the vicinity of beta amyloid has been observed (127). CB2R is also involved in the control of microglial activation, with the release of proinflammatory cytokines (128). The onset of psychosis and microglial activation has been linked (129). It may be possible that activation of CB2R by its agonist provides beneficial effects, relieving the symptoms of psychosis by reducing the microglial activation and release of proinflammatory cytokines. A non-psychoactive phytocannabinoid, cannabidiol, acts as a negative allosteric modulator of CB1R and has been shown to reduce psychotic symptoms in schizophrenia (130). Overall, agents modulating ECS may provide therapeutic benefits against psychosis in AD. The relevant preclinical and clinical studies addressing psychosis in AD, along with their principal findings, are detailed in **Tables 1** and **2**.

## 7 Apathy

The prevalence of apathy in AD is remarkably high, affecting approximately 49% of patients on average, with some studies reporting rates as high as 92% in those with severe cognitive impairment (131). This makes apathy the most common neuropsychiatric symptom observed in AD. It is characterized by a significant loss of motivation that impacts behavior, emotional responsiveness, and goal-directed cognition (132). Unlike depression, which may share overlapping symptoms such as reduced activity and social withdrawal, apathy lacks emotional distress and is increasingly recognized as a distinct clinical syndrome (131). The presence of apathy contributes to faster functional decline, greater caregiver burden, and increased risk of institutionalization, underscoring the importance of early identification and targeted intervention (131, 132).

Neuroimaging and behavioral studies have linked apathy in AD to dysfunction in fronto-striatal circuits, particularly involving the anterior cingulate cortex and prefrontal areas. Likewise, diffusion tensor imaging and PET studies further support the involvement of disrupted fronto-subcortical circuits, including white matter tracts like the cingulum bundle and orbitofrontal cortex connections (133). Reinforcing these imaging findings, postmortem studies have shown that apathy severity is associated with increased neurofibrillary tangle burden specifically in the Anterior Cingulate Cortex (ACC), implicating tau pathology in the degeneration of motivational circuits (122, 134).

Recent evidence highlights the role of the ECS in modulating motivation by regulating dopaminergic transmission in key brain regions such as the prefrontal cortex and striatum. In these areas, typically implicated in reward and goal-directed behavior, ECS activity influences dopamine release and shapes the excitatory-inhibitory balance within cortico-striatal circuits (135). In AD, ECS dysfunction may further contribute to apathy by exacerbating neuroinflammation and impairing the regulation of mesolimbic dopamine pathways. CB1 receptor deficits and imbalances in endocannabinoid tone may reinforce motivational withdrawal (136) (**Figure 3**). As both the ECS and dopamine systems decline with age and disease progression, their interaction may represent a key neurobiological target for understanding and potentially treating apathy in AD.

Apathy in AD has been linked to alterations in several neurotransmitter systems, with the dopaminergic, serotonergic, and glutamatergic pathways being the most important for ECS. ECS dysfunction could alter reward signals and the equilibrium of relevant neurotransmitters (137, 138). For instance, modulation of dopamine release through CB1R activation and its interaction with mesolimbic circuits can considerably influence apathy**’**s motivational behaviors and manifestations (139). The lack of initiative seen in patients with AD could be associated with the reduction of dopaminergic transmission, combined with an alteration in serotonin levels (140). Preclinical studies have shown that cannabinoids can reverse neurotransmitter imbalances observed in AD animal models, suggesting that ECS modulation can restore synaptic function and associated symptoms (141). At the same time, CB1R activation inhibits excitatory neurotransmitter release (such as glutamate), thereby protecting against excitotoxicity, which influences neurodegeneration and the onset of adverse behavioral symptoms (142).

In the same way, as CB2R activation promotes the release of neurotrophic factors and other anti-inflammatory cytokines, its modulation may promote a restoration of the balance between neuroinflammatory and neuroprotective processes, and reduce the damage that affects neuronal plasticity, which is crucial for preserving neuronal integrity and improving behavioral symptoms in AD (51, 75, 76). The integration of these processes, which include the inhibition of NF-κB and the reduction of oxidative stress and other proinflammatory mediators, is presented as a determining factor for the preservation of neuronal function and for the mitigation of behavioral symptoms (14, 51). The convergence of research in neurobiology, neuroimmunology, and molecular pharmacology suggests that targeting the ECS may not only slow synaptic loss in AD but also alleviate behavioral and motivational deficits associated with apathy. Modulating the ECS appears to restore homeostasis in neuronal and immune signaling pathways, potentially reducing apathy and improving overall functional status in AD patients (143). In observational studies, a partial agonist of CB1R and CB2R, derived from cannabis, such as delta-9 THC, has been found to reduce apathy in AD patients (14, 116) (**Figure 3**). **Table 1** provides an overview of preclinical research on apathy in AD, which serves as the basis for the present investigation.

## 8 Disinhibition

In AD, disinhibition manifests in approximately 30% of patients within three years of diagnosis, often presenting as euphoria or socially inappropriate actions (144). Some authors distinguish between social disinhibition (violations of social tact or personal boundaries) and behavioral disinhibition, such as compulsive stealing or utilization behaviors, each potentially linked to distinct neural circuits. Others differentiate emotional disinhibition, characterized by excessive or maladaptive emotional expression, from behavioral forms (145). Additionally, the concept of cognitive inhibition, the suppression of internal or external distractions, is sometimes contrasted with behavioral disinhibition, specifically involving difficulties in regulating emotional and social behaviors within a particular context (146). Impulsivity is often considered a core subcomponent of disinhibition, as reflected in measures such as the NPI. In neurodegenerative disorders such as frontotemporal dementia (FTD), disinhibited behaviors may resemble symptoms of mania or antisocial conduct, sometimes leading to misdiagnosis or legal issues. This cluster of symptoms has been referred to as **‘**acquired sociopathy,**’** particularly in cases involving damage to ventromedial prefrontal regions, including the orbitofrontal cortex (OFC) and anterior cingulate cortex (ACC) (e.g., rostral and subgenual areas) (147, 148).

Pharmacological interventions for disinhibition in dementia are limited and often used when non-pharmacological approaches are ineffective or symptoms are severe. The most commonly employed pharmacological classes include atypical antipsychotics, antidepressants (particularly selective serotonin reuptake inhibitors, SSRIs), anticonvulsants, analgesics, and anti-dementia drugs such as acetylcholinesterase inhibitors and memantine (149). Although atypical antipsychotics such as risperidone, olanzapine, and haloperidol are frequently prescribed, only risperidone is approved for short-term use in AD (150). However, its use is constrained by serious side effects, including an increased risk of stroke and pneumonia (151). Acetylcholinesterase inhibitors and memantine are commonly used for their modest benefits on behavioral symptoms like depression and apathy in dementia, but show no significant effect on disinhibition (152, 153). Analgesics and anticonvulsants, such as valproate, have been explored for behavioral symptoms under the assumption that untreated pain may worsen them; however, evidence supporting their efficacy for disinhibition is lacking (154). While analgesics may reduce general agitation, they do not specifically impact disinhibition. Anticonvulsants offer limited benefit for BPSD and are associated with serious adverse effects, leading to some guidelines advising against their use.

Even though the ECS has not been directly addressed in studies specifically targeting disinhibition in dementia, its known role in modulating emotional regulation, impulsivity, and neuroinflammation suggests that it could represent a promising therapeutic target (155, 156). Few observational studies support that CB1R agonists derived from cannabis significantly reduce disinhibition. One open-label pilot study investigated the addition of a THC-based oil to standard pharmacological regimens in individuals with AD. Over four weeks, significant improvements were observed not only in global NPS but also in specific domains, such as disinhibition, as measured by the NPI. The intervention was generally well tolerated, with only minor and transient adverse events reported (14). Another prospective cohort study evaluated a cannabis-based magistral formulation with a high-CBD/low-THC ratio (50:1) in AD patients with untreated NPS (**Figure 3**). The intervention resulted in a sustained and significant reduction in symptom severity across multiple behavioral domains, including disinhibition, as well as a notable reduction in caregiver distress over a follow-up period of up to 24 months (114). The therapeutic effects are likely attributable to the synergistic interaction between CBD and THC, a phenomenon often referred to as **“**entourage effect**”**, which involves modulation of the ECS, including CB1R and CB2Rs, as well as other targets such as serotonin and TRPV1 receptors.

## 9 Eating disorders

Eating disorders are a common yet often overlooked sign of NPS in AD. These issues can range widely, from anorexia and weight loss to increased food intake and changes in eating habits. In the later stages of AD, it’s especially common for individuals to refuse food, have a reduced appetite, and experience significant weight loss, which can lead to cachexia, a condition marked by frailty, muscle wasting, and a poor response to nutritional support (157). These symptoms aren’t just a natural part of aging or other health issues; they indicate serious neurobiological changes that affect the brain’s appetite-regulating systems and overall metabolic signals (158).

There are several interconnected factors that contribute to eating disorders in AD. It is shown that CB1Rs in the brain have a direct role in modulating both food intake and glucose metabolism, reinforcing the importance of this area in ECS-mediated appetite regulation (159). Neurodegeneration in the hypothalamus and nearby brain areas disrupts the balance of hunger and satiety. Damage to brain key regions interferes with the signals that tell us when to eat or stop eating via orexigenic and anorexigenic pathways (157). This situation is worsened by systemic inflammation, particularly with increased levels of cytokines such as IL-1β and TNF-α, which suppress feeding behavior by altering hypothalamic responses (157). Additionally, patients often face a reduced sense of smell and taste, side effects from polypharmacy, a lack of focus during mealtimes, failure to recognize edible objects as food, and behavioral issues like agitation or apathy, all of which make it even harder to maintain proper nutrition (12, 47).

Weight fluctuations are also linked to the progression of dementia. While being overweight in midlife has been associated with a higher risk of developing AD, likely due to factors like insulin resistance, oxidative stress, and chronic inflammation, anorexia and unintentional weight loss become significant concerns in the later stages of AD and have been linked to faster cognitive decline and increased mortality (47, 160, 161).

The ECS is crucial for regulating feeding behavior, energy homeostasis, and metabolic integration. Under physiological conditions, levels of endocannabinoids such as 2-AG rise during fasting and decline after feeding, accenting the ECS’s role in modulating hunger and satiety states (162). The CB1R, primarily located in the CNS, particularly in areas like the hypothalamus, hippocampus, and limbic regions, functions as an internal regulator. This receptor is essential for controlling appetite and reward-based feeding. When CB1R is activated, the receptor promotes food intake, enhances palatability, and contributes to the hedonic drive to eat (159). Recent experimental studies have elucidated the neurochemical processes that show how CBR affects feeding behavior (163). It turns out that when CB1Rs in the paraventricular nucleus (PVN) of the hypothalamus are activated, they can suppress the release of 5-HT. This reduction then leads to a disinhibition in GABAergic transmission, a pathway that stimulates food intake. This dual action of reducing anorexigenic signals (via 5-HT1A and 5-HT1B receptors) and enhancing orexigenic output highlights the PVN’s role in regulating appetite through the endocannabinoid system. While the study was conducted in healthy rats, these findings raise the possibility that targeted CB1R modulation could have therapeutic implications in conditions such as AD, where hypothalamic dysfunction may contribute to anorexia (163).

In addition to its central effects, ECS activation also promotes food intake by interacting with key peripheral hormones. It increases ghrelin levels, the hunger-stimulating hormone, and decreases leptin, which normally signals satiety. These interactions further illustrate the ECS’s integrative role in appetite control (164). On the flip side, blocking CB1R with an antagonist ligand, like Rimonabant^®^, has been shown to reduce appetite and help with weight loss in obesity studies, but it also came with some serious side effects, including anxiety and depression, which ultimately led to it being pulled from the market (165).

In AD, the ECS can become dysregulated. Research indicates that CB1R expression is altered in brain regions that affect cognition and behavior, such as the hippocampus and prefrontal cortex. In animal studies, lower levels of CB1R have been linked to increased aggression and behavioral issues (13). Particularly in hypothalamic nuclei, reduced CB1R may play a role in causing anorexia and disrupting the normal control of feeding. Therapeutically, cannabinoid agonists show promise to counteract anorexia in dementia patients. Clinical trials with dronabinol (a synthetic form of Δ9-THC) have shown positive results, including improved appetite, weight gain, and a decrease in disturbed behaviors like agitation (47). For instance, in a 12-week observational study, treating 30 AD patients with a THC: CBD oil extract and noted significant enhancements in appetite, weight, sleep quality, and behavioral symptoms (**Figure 3**). The Neuropsychiatric Inventory Questionnaire (NPI-Q) revealed lower scores for agitation, apathy, irritability, and eating issues, while the burden on caregivers also saw a notable decrease (13).

CB1Rs and CB2Rs, which are primarily found in glial cells and immune tissues, have emerged as key players in neuroinflammation. They might also have an indirect effect on feeding behaviors via their impact on neuroimmune crosstalk. Activation of CB2 receptors, primarily expressed in microglia, shifts these cells toward an anti-inflammatory phenotype by suppressing pro-inflammatory cytokines such as IL-1β and TNF-α in response to stimuli such as LPS or IFN-γ (166, 167). Since elevated cytokines drive sickness-related anorexia in AD, CB2R-mediated immunomodulation offers a probable pathway to ameliorate appetite loss indirectly. Additionally, recent studies have identified CB2R orexin receptor heteromers, particularly the CB2R–OX1R complex, as promising targets for managing appetite suppression associated with inflammation in AD. In murine models, blocking OX1R enhanced CB2R signaling, helping restore cyclic AMP (cAMP) responses and reduce neuronal damage (159). The orexin system, particularly orexin-A and its receptor OX1R, has a complex role in both feeding and arousal. When orexin signaling is dysregulated, it can lead to hyperphagia, disrupted sleep patterns, and cognitive impairment. The interaction between orexigenic and cannabinoid pathways provides a useful framework for tackling the mixed symptoms often seen in dementia, especially when agitation, weight loss, and insomnia occur together (159).

Preclinical research also supports the metabolic benefits of cannabinoids. While CBD doesn’t directly activate CB1Rs, it exerts anxiolytic and anti-inflammatory effects through 5-HT1A and TRPV1 receptors, which are relevant in the context of NPS associated with dementia (162). Preclinical research suggests that endocannabinoid signaling can reduce anxiety and promote food intake, particularly through modulation of CB1R in areas like the amygdala. The connection between the neural circuits that regulate anxiety and those involved in appetite control is particularly important, suggesting that interventions aimed at reducing anxiety in dementia patients may have secondary benefits on feeding behavior (155, 162). This supports the rationale for exploring cannabinoids like CBD in the management of anxiety-induced anorexia in neurodegenerative disorders. Considering the connection between the neural circuits that manage anxiety and those that control appetite, anxiolytic interventions such as CBD may indirectly promote feeding in dementia patients, particularly those exhibiting distress-related anorexia.

It is important to recognize that weight loss in dementia is not just a clinical issue; it also serves as a key indicator of prognosis. Long-term studies have revealed that patients who lose more than 5% of their body weight within six months face a greater risk of being placed in care facilities, experiencing cognitive decline, and even increased mortality (168). This highlights the urgent need for targeted interventions, especially in patients with rapid or idiopathic weight loss. The ECS stands out as a modifiable target for treatment, particularly since standard appetite stimulants often fail to meet the needs of these patients. **Tables 1** and **2** summarize the major findings from preclinical and clinical research on eating disorders in AD, including evidence on therapeutic efficacy.

## 10 Discussion

Growing evidence indicates that neurotransmitter imbalances (29), increased neuroinflammation (30, 80), and disease progression play a critical role in behavioral disturbances in AD (32) (**Figure 2**). Behavioral disturbances may partly be due to Tau pathology rather than Aβ deposition (34, 35). Emerging evidence suggests that brain inflammation in dementia follows a biphasic pattern. Initially, microglia are activated in a protective manner, attempting to clear Aβ fibrils through phagocytosis. However, this initial response diminishes over time as microglial function becomes impaired. Subsequently, as tau pathology accumulates, a second phase of heightened microglial activation emerges, characterized by a neurotoxic phenotype that actively contributes to disease progression (39, 169, 170). A preclinical study supports that mice lacking CB1R exhibit heightened aggression, which can be significantly reduced through treatment with CB1R agonists such as Arachidonyl-2’-chloroethylamide (ACEA). These findings indicate a critical role for CB1R in regulating aggression and social behavior (13). Notably, decreased CB1R activity in the hippocampus has also been reported in individuals with AD, suggesting a potential connection to NPS [19]. The NLRP3 inflammasome plays a key role in AD-related neuroinflammation and has been linked to aggression in animal models (80). THC, a partial agonist of CB1R, inhibits NLRP3 activation *in vitro* (171), suggesting its potential to reduce aggression through anti-inflammatory mechanisms similar to those of CB1R agonists. Research indicates that disruptions in GABAergic and serotonergic neurotransmission are associated with increased agitation in individuals with AD (33). Studies have demonstrated that cannabinoid agonists stimulate [³H] GABA release through activation of CB1R in rats (44). Additionally, CB1R knockout mice show impaired function of 5-HT1A and 5-HT2A/C receptors, indicating a regulatory role for CB1R in serotonergic signaling. These findings suggest that CB1R activation may enhance GABA release and stabilize serotonergic receptor function without altering overall 5-HT levels (172). As a partial CB1R agonist, delta-9 THC may offer therapeutic benefits in managing agitation.

ECS plays a crucial role in regulating sleep. A high prevalence of sleep disturbances was noticed in AD patients (173). A partial agonist of CB1R was found to be beneficial in reducing sleep disturbances (14). A preclinical study has revealed that stimulation of the CB1R induces sleep, an effect that selective CB1 receptor antagonists effectively inhibit (174). Further studies demonstrate that loss of CB1R results in multiple sleep alterations (175).

An open-label prospective study evaluated the safety and efficacy of non-psychoactive cannabinoid, CBD-rich oil, for NPS in AD. Among 59 patients treated for a mean of 23.2 months, CBD significantly reduced NPI-Q severity and caregiver distress scores, with 94.9% achieving more than 30% improvement and over half showing more than 50% reduction. The findings suggest that CBD-rich oil is a safe and effective long-term therapy for managing NPS and alleviating caregiver burden in AD (116). However, the therapeutic onset is longer with CBD compared to psycho active compound THC. FAAH and MAGL inhibitors, which elevate endogenous cannabinoid tone by blocking endocannabinoid degradation, offer a safer alternative to direct CBR agonists. High doses of CBR agonists may cause undesirable side effects due to nonselective binding to other receptors or impair neuronal signaling. Whereas ECS Inhibitors of the ECS enzymes FAAH and MAGL, which can elevate endocannabinoid levels indirectly and may offer therapeutic benefits against depression and anxiety with fewer side effects. Preclinical studies have demonstrated that FAAH and MAGL inhibitors help regulate the HPA axis, support synaptic plasticity, reduce amyloid-β accumulation, protect dopaminergic neurons, and counteract neuroinflammation (74). Currently, an FAAH and MAGL inhibitor is under investigation to evaluate its safety, tolerability, and efficacy for the treatment of agitation in AD (NCT06808984). However, low doses of CB1R, a partial agonist, both synthetic, dronabinol, and natural, cannabis derived delta-9 THC, are found to be beneficial in treating agitation, depression, anxiety, nighttime sleep problems, and hallucinations in AD patients (14, 47–49).

### 10.1 Limitation

Several preclinical and clinical studies demonstrate that ECS plays a role in many neuropsychiatric conditions. The agents that modulate the ECS have beneficial effects in managing many NPS in AD. However, to date, no FDA-approved ECS-based drug has been approved to treat NPS in AD. Chronic activation of CB1R has been shown to desensitize receptor function in preclinical studies. High doses of cannabinoids can cause memory deficits by excessively occupying CB1Rs, disrupting the precise regulation of endocannabinoid-driven synaptic plasticity, as noted by Carlson et al. (176). In the general population, preserved CB1R function makes higher THC doses more likely to impair cognition. In contrast, the CB1R loss observed in AD patients suggests that carefully controlled CB1R activation could offer therapeutic benefits (29). An individual with the cytochrome P450 2C9 polymorphism affects THC metabolism (177). More placebo-controlled studies are warranted to understand the chronic effects of cannabinoid therapies on cognitive impairments, drug efficacy, and other side effects.

### 10.2 Heterogeneity in NPS

NPS in AD can manifest in three different phases: (1) as premorbid risk factors that precede the onset of AD pathology, (2) as early or prodromal indicators reflecting emerging neurodegenerative changes, with or without cognitive decline, and (3) as new-onset symptoms arising during the dementia stage as a result of ongoing disease processes (178). Several recent studies indicate that mechanistic heterogeneity in NPS is associated with genetic, environmental, and sex differences (178). The first significant genome-wide loci associated with psychosis in Alzheimer’s disease dementia, SUMF1 and ENPP6, were identified in a cohort of 12,317 cases (179). Additionally, the first differentially methylated genomic regions, located in TBX15 and WT1, were reported, highlighting potential epigenetic mechanisms contributing to neuropsychiatric manifestations in AD (180). Transcriptomic studies in AD have identified gene signatures linked to specific NPS domains, agitation, psychosis, affective symptoms, and apathy, but no single signature spans all four domains, highlighting the need for replication and functional validation (181). A meta-analysis of over 21,000 patients found that women with AD show higher prevalence and severity of depression, psychosis, and aberrant motor behavior, while men exhibit more severe apathy; no sex differences were observed for other NPS (182). A recent study in dementia patients, logistic regression analyses revealed that distinct NPS subtypes were differentially associated with neuropathologic features, early psychosis linked to Lewy body and white matter pathology, while late psychosis correlated with AD-related lesions, including neurofibrillary tangles, neurotic plaques, and cerebral amyloid angiopathy. Overall, these findings suggest that the timing and nature of NPS reflect specific neuropathologic processes, which may inform targeted therapeutic interventions (183). Despite heterogeneity in NPS, no precision treatment has been developed. Based on observational studies with modulators of the ECS system for NPS in AD, beneficial effects have been observed in various domains. More placebo-controlled studies are warranted to understand the chronic effects of cannabinoid therapies on cognitive impairments, drug efficacy, and other side effects.

## 11 Conclusion

ECS is essential for regulating a range of behaviors and undergoes notable changes during the progression of AD, particularly through modulation of the CB1R and CB2Rs and their endocannabinoids. Further placebo-controlled, randomized clinical trials are needed to confirm the efficacy of cannabinoid receptor-targeting therapies in managing NPS in AD.
